# Case Report: A rare case of hepatic undifferentiated pleomorphic sarcoma: diagnostic dilemmas and multidisciplinary management strategies

**DOI:** 10.3389/fonc.2025.1572348

**Published:** 2025-08-06

**Authors:** Yu Yang, Yiwei Hou, Li Yi, Mingzhi Jiang, Lihua Tang, Mingzheng Hu, Rongchun Xing

**Affiliations:** ^1^ The First College of Clinical Medical Science, China Three Gorges University, Yichang, Hubei, China; ^2^ Department of Hepatobiliary Surgery, Yichang Central People’s Hospital, Yichang, Hubei, China; ^3^ Department of Endocrinology, Yichang Central People’s Hospital, Yichang, Hubei, China; ^4^ Medical Technology College of Qiqihar Medical College, Qiqihar, Heilongjiang, China; ^5^ Department of Hepatobiliary and Pancreatic Surgery, Qiaokou District People’s Hospital, Wuhan, Hubei, China; ^6^ Department of Pathology, Yichang Central People’s Hospital, Yichang, Hubei, China

**Keywords:** undifferentiated pleomorphic sarcoma, liver tumour, rare malignancy, case report, multidisciplinary management

## Abstract

Undifferentiated pleomorphic sarcoma of the liver is an extremely rare mesenchymal malignancy, and there is limited literature on its clinical manifestations, diagnostic challenges, and therapeutic strategies. The case is unique due to its large tumor size, encapsulated feature, and lack of vascular invasion, offering new insights into tumor characteristics. This case was documented to contribute to the understanding of this aggressive tumor and highlight the role of multidisciplinary management in achieving favorable outcomes. A 57-year-old female patient presented with persistent upper abdominal pain for over 2 months. Laboratory tests showed anemia, hypoalbuminemia, and elevated AFP, and imaging revealed a large hepatic mass with necrosis. Initially, imaging misdiagnosed the large hepatic mass as hepatocellular carcinoma, but subsequent pathology and immunohistochemistry confirmed it was undifferentiated pleomorphic sarcoma. The patient successfully underwent left hepatic lobectomy with clear margins, and the resected tumor was a high-grade spindle cell tumor with significant necrosis. Postoperatively, the patient recovered well and was discharged in stable condition, with recommendations for further molecular diagnostics and oncological follow-up. In conclusion, early surgical intervention and multidisciplinary approaches are crucial for managing rare and aggressive hepatic tumors, such as undifferentiated pleomorphic sarcoma.

## 1 Introduction

Undifferentiated pleomorphic sarcoma (UPS), formerly known as malignant fibrous histiocytoma, is a rare, aggressive mesenchymal tumor with poorly understood pathogenesis and a predilection for the extremities and retroperitoneum (1, 2). Its occurrence in the liver is exceedingly uncommon, presenting unique diagnostic and therapeutic challenges due to its rarity, nonspecific clinical manifestations, and aggressive biological behavior. This case report highlights a rare presentation of hepatic UPS with a massive tumor burden, emphasizing the significance of multidisciplinary approaches in diagnosis and management.

The purpose of this case report is to illustrate the clinical, radiological, and pathological features of hepatic UPS and to discuss its diagnostic and therapeutic implications in light of existing literature. Given the lack of established guidelines for hepatic UPS, this report aims to contribute to the growing body of knowledge on this rare malignancy and to inform clinical decision-making in similar scenarios.

A comprehensive literature review was conducted using PubMed and Web of Science databases. The search terms included “undifferentiated pleomorphic sarcoma,” “hepatic sarcoma,” “liver tumor,” and “rare hepatic malignancies.” Relevant articles were reviewed to contextualize the rarity of this case, compare clinical and pathological features, and identify gaps in knowledge regarding its management (**Table 1**). The findings underscore the merit of documenting and sharing unique cases like this, as they provide insights into rare disease processes and highlight the need for further research and standardized treatment approaches.

**Table 1:** Clinical, imaging, pathological, and treatment features of primary hepatic undifferentiated pleomorphic sarcoma.

Study	Patient demographics	Clinical presentation	Imaging findings	Pathology and immunohistochemistry	Treatment and outcome
Wang Z et al. (2023) (3)	7 patients	Asymptomatic masses found during screening	CT, MR: large, solitary, non-encapsulated mass, often with hypoechoic or heterogeneous echogenicity	Pathology: pleomorphic spindle cells, SMA+, vimentin+, desmin-, HMB-	Surgery not described, imaging diagnosis aimed at differential diagnosis
Pellegrini JR et al. (2022) (4)	56-year-old male	Epigastric abdominal pain (6 months)	CT: numerous hepatic masses involving both liver lobes	Pathology: spindle cell neoplasm, SMA+, focal h-caldesmon+, negative for desmin and others	CT-guided biopsy confirmed the diagnosis; chemotherapy was rejected
Sun H et al. (2024) (5)	59-year-old male	Asymptomatic mass detected on screening	CT, MR: 5-cm mass with mild peripheral enhancement	Pathology: vimentin+, SMA+, *Ki67*+ (50%+), negative for epithelial markers	Partial hepatectomy, chemotherapy (gemcitabine, karelizumab), recurrence within 6 months
Suzuki H et al. (2024) (6)	60-year-old female	Rapidly growing tumor, inoperable	CT, MR: tumor growth, no specific enhancement	Pathology: vimentin+, SMA+, *KRAS*, and *TP53* mutations found	Hepatic arterial infusion chemotherapy, the tumor continued to grow, and the patient died 8 months post-symptom onset
Mass JB et al. (2018) (7)	56-year-old male	Abdominal pain, jaundice, history of cholecystitis	CT, PET: Hypermetabolic mass in the liver	Pathology: pleomorphic spindle cells, negative for epithelial and hematolymphoid markers, vimentin+	Chemotherapy (doxorubicin, ifosfamide); the patient died 19 days post-diagnosis

This table compiles primary hepatic UPS case reports, detailing demographics, presentation, imaging, pathology, immunophenotype, therapy, and outcomes. Patients are usually asymptomatic or report vague abdominal pain. Imaging shows large non-encapsulated, hypoechoic/heterogeneous masses with peripheral enhancement. Histology reveals pleomorphic spindle cells; immunostaining is vimentin- and SMA-positive, and epithelial/neural/hematologic markers are negative. Surgery remains first-line but recurrences are frequent; chemotherapy provides limited benefit. Inoperable disease or tumors with KRAS/TP53 mutations portend poor survival, underscoring diagnostic complexity and the need for more effective treatments.

This case involves a 57-year-old female patient presenting with a massive hepatic tumor initially diagnosed as hepatocellular carcinoma and later confirmed to be UPS through histopathological and immunohistochemical analyses. Notably, the tumor’s large size (23.5 cm), its encapsulated nature despite extensive necrosis, and the lack of vascular invasion or satellite nodules are highly unusual in hepatic UPS cases, further highlighting its uniqueness. The rarity of the condition, combined with its unique clinical and pathological features, renders this case particularly valuable for advancing the understanding and management of hepatic mesenchymal malignancies.

## 2 Case presentation

A 57-year-old female patient presented with persistent upper abdominal pain lasting for over 2 months. The pain was described as continuous and dull, associated with fatigue, poor appetite, and occasional nausea without vomiting. The patient denied experiencing fever, chills, cough, chest discomfort, dyspnea, urinary symptoms, or other systemic complaints. She sought medical attention at a local hospital, where abdominal enhanced computed tomography (CT) showed a massive tumor in the left lobe of the liver with features suggesting malignancy. Her serum alpha-fetoprotein (AFP) was elevated to 810.3 ng/mL. Under local anesthesia, she underwent hepatic artery embolization and chemotherapy perfusion. Postoperatively, supportive treatments including liver protection and symptomatic management improved her condition, and she was discharged. However, the abdominal pain persisted intermittently, accompanied by reduced food intake. The patient was admitted to this hospital for further evaluation and treatment.

She had no significant past medical history, no prior surgeries, no known allergies, and no history of blood transfusions. She denied smoking, alcohol consumption, or drug use. Her vaccination history was unremarkable. The patient’s medication history included supportive liver therapy post-procedure but no long-term prescribed medications. She was a resident of a non-endemic area, working locally in an undefined occupation. Her family history was unremarkable, with no known familial diseases, malignancies, or genetic disorders. There was no reported significant psycho-social stressor affecting her health recently.

On physical examination at admission, the patient was alert and oriented. Her vital signs were stable: blood pressure, 125/91 mmHg; heart rate, 88 bpm; respiratory rate, 20 breaths per minute; and temperature, 36.6°C. A detailed timeline of diagnosis and treatment and findings is shown in **Table 2**. Her sclera was non-icteric. An abdominal examination revealed a palpable firm mass under the xiphoid process, immobile on palpation, with no rebound tenderness or abdominal wall varices. No hepatomegaly, splenomegaly, or ascites was noted. The lower extremities showed no edema, and the neurological examination was unremarkable.

**Table 2:** Timeline of care for patient.

Date	Event	Findings
2024.11.6	First diagnosis at Wufeng County Hospital	Upper abdominal enhanced CT: large mass in the left lobe of the liver, suspected tumor with stroke; AFP: 810.3 ng/mL
2024.11.8	Treatment at Wufeng County Hospital	Underwent liver artery angiography + chemoembolization under local anesthesia
2024.12.09	Admission to Yichang Central People’s Hospital	Complaint of persistent upper abdominal pain for over 2 months; various examinations arranged
2024.12.10 - 2024.12.12	Multiple tests at Yichang Central People’s Hospital	Blood tests, CT, MR, PET/CT, etc., showing liver tumor-related changes, possible right-sided adnexal mass, and other findings
2024.12.16	Surgery at Yichang Central People’s Hospital	Resection of left lateral lobe of liver tumor. The pathological diagnosis was high-grade sarcoma with necrosis, which tended to be undifferentiated pleomorphic sarcoma.
2024.12.18 - 2024.12.22	Post-operative tests at Yichang Central People’s Hospital	Blood and biochemical tests to monitor recovery.

Timeline of care initial CT and elevated AFP at Wufeng County Hospital; hepatic arterial embolization; admission to Yichang Central People’s Hospital with comprehensive laboratory and imaging work-up; left hepatectomy achieving R0 margins, pathology confirming undifferentiated pleomorphic sarcoma; postoperative biochemical and imaging surveillance.

The laboratory tests on admission showed anemia with hemoglobin of 96 g/L and hypoalbuminemia with serum albumin at 31.6 g/L. C-reactive protein was elevated at 101.51 mg/L. The liver function tests revealed mildly elevated alanine transaminase (74 U/L) and aspartate transaminase (176 U/L). The AFP remained markedly elevated at 1,092 ng/mL. Coagulation studies indicated a fibrinogen level of 4.84 g/L and normal prothrombin time. The blood and urine cultures were negative, and viral serologies ruled out hepatitis B and C infections.

**Figure 1:**
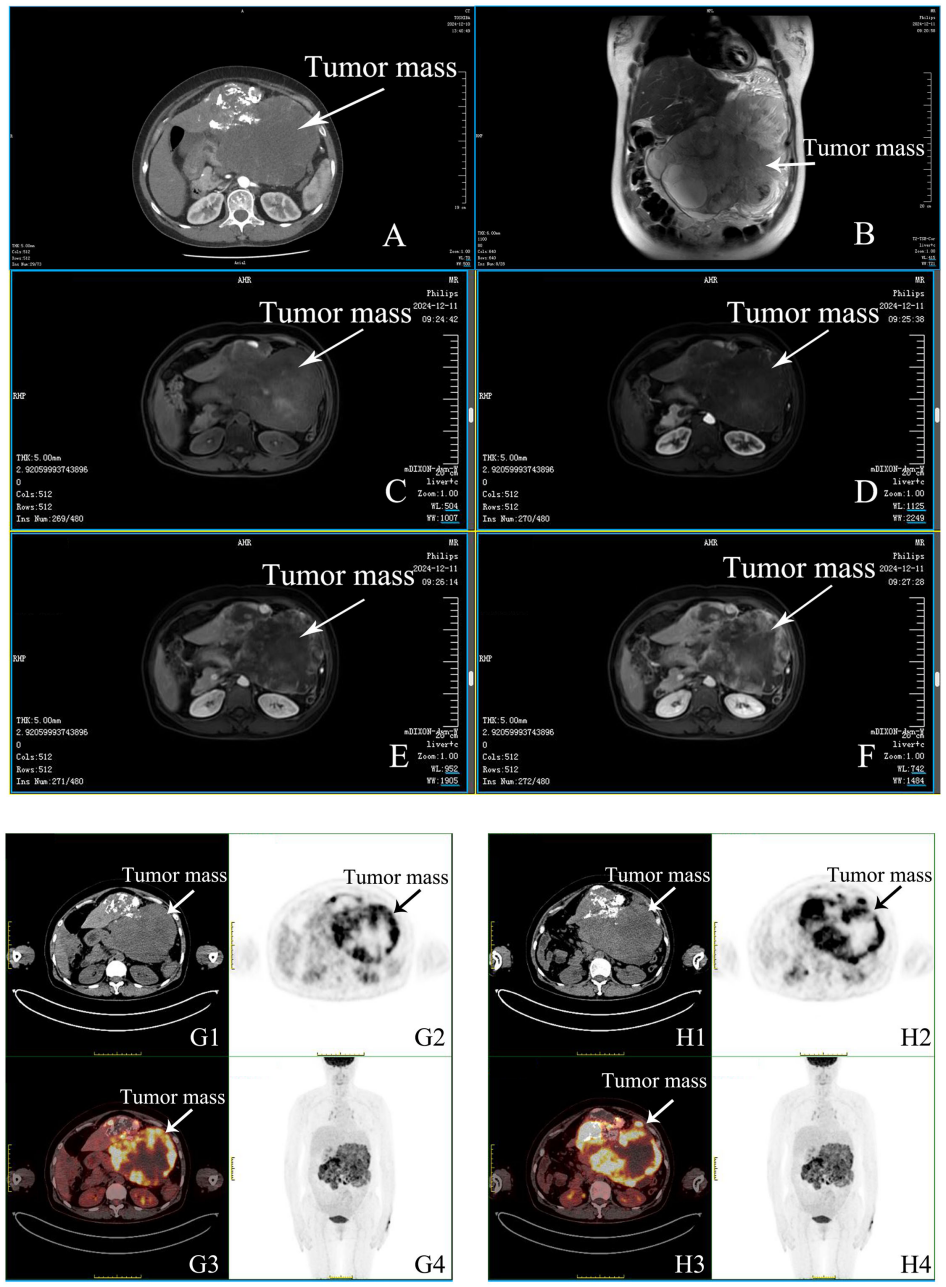
Imaging findings of post-treatment hepatocellular carcinoma with associated complications. **(A)** Contrast-enhanced CT (CTA, CTV) demonstrates post-interventional changes in the liver. The left hepatic lobe shows a large heterogeneous, hypodense mass extending into the abdominal cavity (22.4 × 12.6 cm). The mass contains multiple hypodense areas with iodized oil deposition. Arterial phase imaging shows peripheral enhancement, while portal and delayed phases show hypodensity relative to the surrounding liver parenchyma. Additional round, non-enhancing foci are observed within the liver. Normal biliary structures are noted. Splenic vein stenosis and possible right adnexal mass with pelvic effusion are evident. **(B)** Axial T2-weighted MRI reveals a large mixed-signal mass in the left hepatic lobe (22.9 × 13.7 cm) protruding into the abdominal cavity. The lesion shows multiple long T1 and T2 signal areas suggestive of necrosis or fluid. **(C–F)** Axial mDIXON MRI sequences show the liver lesion with heterogeneous signal intensities. Arterial phase enhancement highlights active tumor areas, while non-enhancing regions correspond to necrotic or cystic components. Associated findings include a hepatic cyst and adjacent vascular and intestinal displacement. **(G1–H1)** Axial CT scans of the abdomen show a large exophytic mass in the left hepatic lobe (22.1 × 13.9 cm), containing mixed densities with iodized oil deposition and necrotic areas. Mildly enlarged lymph nodes are observed in the mediastinum (SUVmax 5.5). **(G2–H2)** PET scans display regions of increased metabolic activity within the hepatic lesion, with a maximum SUV of 17.4, indicating residual tumor activity. **(G3–H3)** Fused PET/CT images highlight the precise localization of metabolic activity within the hepatic mass and mediastinal lymph nodes, combining anatomical and functional data. **(G4–H4)** The whole-body PET scan shows no evidence of distant metastasis. Background uptake is normal in the brain, thyroid, and other major organs. Moderate pelvic effusion and right adnexal mass (3.8 × 2.1 cm) are seen, with low metabolic activity. Additional mild uptake (SUVmax 2.3) is noted in a small nodule in the left upper lobe of the lung, suggesting an inflammatory process.

Imaging studies included an abdominal enhanced CT that confirmed a large left hepatic lobe tumor (22.4 cm × 12.6 cm) with heterogeneous enhancement and features suggestive of necrosis. Positron emission tomography–computed tomography (PET-CT) demonstrated intense metabolic activity in the hepatic mass (maximum standardized uptake value (SUVmax), 17.4) and identified a metabolically active nodule in the left lung (SUVmax, 2.3), likely inflammatory. The finding of magnetic resonance imaging (MRI) corroborated the CT findings, showing a heterogeneous mass with arterial enhancement and necrotic areas (**Figure 1**).

**Figure 2:**
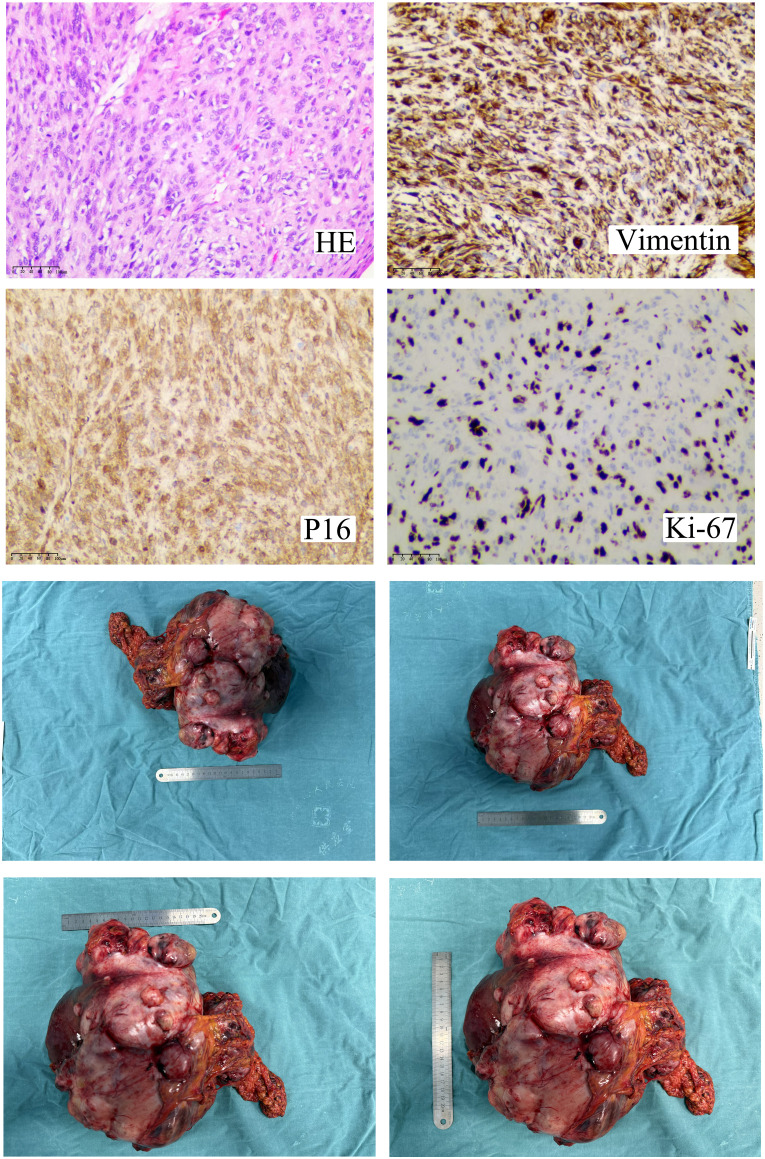
Histopathological, immunohistochemical, and gross features of the left-lobe undifferentiated pleomorphic sarcoma. Hematoxylin–eosin staining shows a high-grade spindle-cell proliferation arranged in intersecting fascicles with a “fish-bone” pattern, foci of coagulative necrosis, and scattered pleomorphic giant cells. Immunoprofiling reveals diffuse vimentin and strong p16 expression, while ~60% of the tumor nuclei are Ki-67-positive, indicating rapid proliferation. The resected specimen is an encapsulated nodular mass measuring 27 × 25.5 × 12 cm, sharply demarcated from the surrounding parenchyma. Its cut surface is soft, gray-white to gray-yellow with focal cystic, mucin-filled spaces; gross hemorrhage, vascular invasion, and satellite nodules are absent. The combined findings confirm a high-grade mesenchymal malignancy compatible with undifferentiated pleomorphic sarcoma.

Surgical exploration was performed, and a left hepatic lobectomy was carried out. Intraoperatively, the tumor was found to be encapsulated, measuring 23.5 cm × 14.5 cm × 9.6 cm, with no gross vascular invasion or satellite lesions. The resection margins were clear. The pathological examination revealed a high-grade spindle cell sarcoma with necrosis. The tumor exhibited a characteristic “fishbone” pattern and included giant tumor cells. Immunohistochemistry was significant for *Ki-67* expression in 60% of tumor cells, positive staining for vimentin and *P16*, and negative staining for hepatocyte markers (arginase-1 and hepatocyte). These findings were consistent with undifferentiated pleomorphic sarcoma (**Figure 2**).

Postoperatively, the patient recovered well, with supportive treatments including nutritional therapy and pain management. The patient shared that she was relieved to have the tumor removed. She found the postoperative supportive treatments helpful in managing her discomfort. Despite some concerns about the long-term prognosis, she was positive about following the recommended follow-up plan for better health. However, the details of potential adjuvant therapies such as chemotherapy or radiotherapy, including dosage, strength, and duration, remain undetermined and need further exploration in future studies. She was discharged on postoperative day 10 in stable condition. The patient reported feeling better in terms of abdominal pain and appetite, but her self-assessment of long-term well-being remains to be seen during follow-up. She was advised to undergo further molecular diagnostics and consider systemic therapy based on multidisciplinary recommendations. The assessment of the patient’s adherence to and tolerability of the recommended interventions will be evaluated through regular outpatient follow-up interviews and monitoring of adverse reactions. The follow-up included serial AFP measurements, imaging studies, and consultation for adjuvant therapy.

At the 6-month postoperative follow-up, the patient underwent enhanced CT imaging and serum AFP monitoring. Repeat abdominal CT demonstrated no evidence of local recurrence or distant metastasis, with complete resolution of the previously noted pelvic effusion. Serial AFP measurements showed a progressive decline to 12.7 ng/mL, approaching the normal range (0–7 ng/mL). The asymptomatic patient with Eastern Cooperative Oncology Group performance status 0 has been determined not to require adjuvant chemotherapy or radiotherapy after a comprehensive oncology multidisciplinary team evaluation. The oncology multidisciplinary team has recommended continued close surveillance with bimonthly clinical evaluations and imaging follow-up.

## 3 Discussion

Undifferentiated pleomorphic sarcoma, an extremely aggressive soft tissue sarcoma, exhibits distinct epidemiological, etiological, and treatment-related characteristics. Epidemiologically, UPS most commonly occurs in the trunk and extremities, yet its incidence in the kidney is extremely rare as reported in some cases (8). It seems to have a predilection for affecting the elderly, with some studies indicating a higher prevalence among this age group (9). The exact etiology of UPS remains unclear. Ultraviolet radiation has been suggested as a potential etiological factor in some cases, especially for those with cutaneous manifestations. In terms of treatment, wide excision is often considered the gold-standard treatment approach when feasible, aiming to remove the tumor with clear margins to reduce the risk of local recurrence. Chemoradiotherapy may be used as an adjunctive therapy—for example, in some cases, chemotherapy with drugs like doxorubicin and ifosfamide has been employed, either alone or in combination, following surgical resection (10). In cases where surgery is not possible due to the extent of the disease or the location of the tumor, such as in metastatic cases, palliative chemotherapy may be the main treatment option to control symptoms and slow down disease progression. However, the prognosis of UPS is generally poor, with high rates of local recurrence and distant metastasis, highlighting the need for continued research to develop more effective treatment strategies.

The presented case, involving a massive hepatic tumor initially misdiagnosed as hepatocellular carcinoma, underscores the challenges in diagnosing and managing this rare entity. This discussion compares the clinical, pathological, and therapeutic aspects of the case with existing literature while addressing its unique features, limitations, and implications for clinical practice.

The patient presented with nonspecific symptoms of abdominal pain, which is consistent with most reported cases of hepatic UPS where vague abdominal discomfort is the predominant symptom. During the diagnostic process, no obvious issues regarding access to testing, financial burden, or cultural factors were reported. Imaging studies showed a large, well-defined, heterogeneous mass with necrotic areas and high metabolic activity on PET-CT, features commonly described in hepatic UPS (7). However, the initial misdiagnosis of hepatocellular carcinoma reflects the clinical challenge posed by the overlapping imaging characteristics of UPS and primary hepatic tumors such as hepatocellular carcinoma and intrahepatic cholangiocarcinoma. Immunohistochemistry played a pivotal role in differentiating UPS, as the tumor was negative for hepatocyte-specific markers (arginase-1 and hepatocyte) and positive for vimentin and *P16*, aligning with diagnostic criteria in the literature (11).

The pathological findings in this case included a high-grade spindle cell morphology with “fishbone” patterns and significant necrosis, consistent with descriptions of UPS in hepatic and non-hepatic locations (12). However, the tumor staging was not clearly defined in this case, which is an important factor affecting prognosis in oncology. The immunohistochemical profile further validated the diagnosis, with a high *Ki-67* proliferation index (60%), indicating aggressive behavior. These features are corroborated by reports of poor prognosis in hepatic UPS, with high recurrence rates and limited long-term survival despite aggressive surgical intervention (4, 13).

The therapeutic approach in this case involved left hepatic lobectomy with clear surgical margins. Surgical resection is the mainstay of treatment for UPS, as highlighted in the literature (14). However, the rarity of hepatic UPS has precluded large-scale studies, leaving a lack of standardized postoperative protocols (4). Based on current research, adjuvant therapy for undifferentiated pleomorphic sarcoma, including chemotherapy and radiotherapy, remains controversial. If the patient’s condition deteriorates or recurrence occurs during follow-up, changes in a therapeutic intervention such as adding chemotherapy or adjusting the intensity of radiotherapy may be considered, depending on the patient’s overall condition and tumor characteristics. While some studies show that adjuvant radiotherapy may reduce local failure risk in extreme cases, the role of chemotherapy is unclear (15). Molecular diagnostics, including targeted sequencing, could provide insights into actionable mutations and guide future therapeutic strategies (16).

The uniqueness of this case lies in the size of the tumor (23.5 cm), the encapsulated nature of the mass despite extensive necrosis, and the absence of vascular invasion or satellite nodules, which are uncommon in such aggressive tumors. These findings challenge the typical presentation of hepatic UPS and highlight the need for individualized management strategies based on tumor biology and patient-specific factors.

The strengths of this case report lie in its unique tumor features, which provide new insights into the characteristics of hepatic UPS, and the emphasis on multidisciplinary management, offering a practical reference for similar cases. The limitations of this report include the lack of molecular analysis to identify potential therapeutic targets and the short follow-up period to evaluate long-term outcomes. These limitations underscore the necessity for further research and case accumulation to better understand the pathogenesis, treatment response, and prognostic factors of hepatic UPS.

The temporal relationship between symptom onset, diagnosis, and treatment progression aligns with existing data on the aggressive course of UPS. The causal relationship between the patient’s clinical presentation and the tumor’s pathological findings is well supported by the diagnostic workup and histopathological confirmation.

This case highlights the diagnostic challenges and therapeutic considerations in managing hepatic UPS, emphasizing the importance of multidisciplinary collaboration and the integration of advanced diagnostic techniques. The scientific rationale for these conclusions lies in the rarity of hepatic UPS, the overlapping imaging features with other hepatic tumors, the aggressive nature of the tumor indicated by a high *Ki-67* index, and the lack of standardized treatment protocols, all of which necessitate a comprehensive approach for better patient outcomes. Recommendations include prioritizing early surgical intervention, considering molecular diagnostics to explore targeted therapy options, and fostering multicenter collaborations to advance the understanding and treatment of this rare malignancy. The rarity and complexity of hepatic UPS necessitate continued documentation of cases to refine diagnostic and therapeutic strategies (17).

## 4 Conclusion

In conclusion, this case highlights the diagnostic and therapeutic complexities of undifferentiated pleomorphic sarcoma of the liver, a rare and aggressive malignancy. Diagnosing hepatic UPS is difficult. Early surgery and multidisciplinary management are crucial. Early recognition through comprehensive imaging and definitive diagnosis via immunohistochemistry are crucial to differentiate UPS from other hepatic tumors. Surgical resection with clear margins remains the cornerstone of treatment, though the role of adjuvant therapy requires further study. This case underscores the importance of a multidisciplinary approach, integrating advanced diagnostics and personalized management strategies, to optimize outcomes. Future research should focus on molecular profiling to identify potential therapeutic targets and better understand the pathogenesis of hepatic UPS. The insights from this case inform the need for vigilance in diagnosing rare hepatic tumors and reinforce the value of individualized treatment plans in clinical practice.

## Data Availability

The raw data supporting the conclusions of this article will be made available by the authors, without undue reservation.
